# Biomass-Derived Carbon Electrodes with Defects and Porosity Prepared via Regulated Carbonization Temperature for Supercapacitors

**DOI:** 10.3390/ma19173741

**Published:** 2026-09-03

**Authors:** Tserenlkham Byambadorj, Jiawei Zhang, Xuzhen Lu, Yuehui Wang, Fan Wang, Qian Liu, Yu Li, Minghua Chen

**Affiliations:** 1 Key Laboratory of Engineering Dielectric and Applications (Ministry of Education), School of Electrical and Electronic Engineering, Harbin University of Science and Technology, Harbin 150080, China; tserenlkham.b@must.edu.mn (T.B.);; 2 School of Power Engineering, Mongolian University of Science and Technology, Ulaanbaatar 14191, Mongolia

**Keywords:** biomass-derived carbon, carbonization temperature, defects, supercapacitor

## Abstract

**Highlights:**

- Activation-free corn straw-derived carbon electrodes were successfully prepared without chemical activating agents or additives.
- Carbonization temperature significantly regulated the pore structure, defect density, carbon microstructure, and electrochemical performance.
- Carbon prepared at 800 °C exhibited the highest electrochemical performance and excellent cycling stability over 30,000 cycles.
- Electrochemical behavior was governed by the synergistic effects of pore characteristics, defect-related surface features, and the carbon microstructure rather than by surface area alone.
- This work provides a simple, sustainable, and scalable strategy for the design of biomass-derived carbon electrodes for practical energy-storage applications.

**Abstract:**

Biomass-derived carbons hold substantial promise for sustainable electrochemical energy storage due to their low cost, wide availability, and intrinsic heteroatom- and mineral-rich nature. However, the fundamental influence of carbonization temperature on the structural evolution of non-activated biomass-derived carbons remains insufficiently understood. In this work, corn straw-derived carbon (CS) is produced without any chemical additives to isolate the intrinsic effects of carbonization temperature on its physicochemical properties. Systematic temperature variation from 600 to 1000 °C reveals pronounced changes in micro-morphology, pore development, defect density, and the ordering of the carbon matrix, all strongly governed by the inherent mineral content of corn straw. Electrochemical evaluation in alkaline electrolyte demonstrates that CS-800 delivers the highest specific capacitance of 53.8 F g^−1^ at 1 A g^−1^ in a three-electrode configuration and maintains favorable rate capability in a symmetric supercapacitor device. The symmetric coin-cell supercapacitor device assembled with CS-800 as the electrodes achieved an energy density of 3.64/5.8 Wh kg^−1^ and a power density of 5200/750 W kg^−1^, along with remarkable cycling stability over 30,000 cycles with negligible capacitance loss. Overall, this study provides mechanistic insight into temperature-driven structural evolution in non-activated biomass-derived carbons, offering a fundamental understanding that may guide the rational design and future development of sustainable carbon electrodes for electrochemical energy-storage applications.

## 1. Introduction

The growing energy crisis and environmental issues associated with fossil fuels have accelerated the transition toward renewable and sustainable energy systems, thereby driving the urgent need for efficient, cost-effective, and long-lifespan rechargeable batteries to meet present and future energy demands [1]. Among the various candidates, supercapacitors (SCs) have attracted significant attention as advanced energy-storage devices owing to their high power density, long cycling life, rapid charge–discharge capability, and environmental friendliness. The performance of SCs is critically dependent on the development of electrode materials with tailored nanostructures, such as hierarchical porosity, high electrical conductivity, abundant electrochemically active sites, and robust structural stability [2,3]. Among these, carbon-based materials—including graphene, carbon nanotubes, carbon nanofibers, mesoporous carbons, and activated carbons—have been widely investigated and remain central to SC research due to their favorable physiochemical properties [4,5].

Recently, biomass-derived carbon materials have emerged as promising alternatives for sustainable electrode materials because of their natural abundance, renewability, and intrinsic heteroatom content [6,7]. A variety of biomass precursors such as coconut shells, bamboo, lignin, agricultural residues, and other plant-derived wastes have been successfully utilized to fabricate porous carbons for supercapacitor applications. For example, activated carbon derived from rubber plant leaf petioles has demonstrated excellent electrochemical performance, confirming the feasibility of agricultural biomass as a carbon precursor [8]. Similarly, carbon materials prepared from Convolvulus arvensis biomass have exhibited effective charge-storage behavior in symmetric supercapacitor systems [9]. However, most of these studies rely on chemical activation or templating agents (e.g., KOH, ZnCl_2_, or H_3_PO_4_) to achieve high surface areas and hierarchical porosity. While effective, such approaches often obscure the intrinsic relationship between carbonization condition and the structural evolution of biomass-derived carbons. In addition, the electrochemical performance of biomass-derived carbon is governed not only by its specific surface area and pore distribution but also by its defect density, surface functional groups, graphitization degree, and microstructural ordering [10,11,12,13,14]. Therefore, understanding how synthesis parameters influence these structural features is essential for rational material design.

Various strategies have been proposed to improve their capacitive performance, including heteroatom doping (e.g., N, S, and O), pore structure engineering, and optimization of heat-treatment conditions, with recent efforts increasingly directed toward developing cost-effective, sustainable, and scalable approaches for large-scale production [15,16,17,18,19]. Among these, carbonization temperature plays a particularly critical role in determining the structural and electrochemical properties of biomass-derived carbon materials. Previous studies have shown that moderate temperatures can promote pore development, whereas excessively high temperatures may lead to pore collapse and reduced surface area. For instance, Qiu et al. [20] reported that increasing activation temperature can decrease pore volume due to structural shrinkage, while Xue et al. [21] observed that higher temperatures induce lattice contraction, as evidenced by diffraction peak shifts. More recently, Lobato-Peralta et al. [22] and Ma et al. [23] emphasized that careful control of carbonization temperature is essential for achieving activated carbons with optimal surface area. Despite these advances, most reported works involve complex processes such as pre-carbonization followed by chemical activation, hydrothermal treatment, or templating strategies. These approaches make it difficult to isolate the intrinsic influence of carbonization temperature on the microstructure and electrochemical behavior of biomass-derived carbons. Consequently, systematic investigations of additive-free carbonization processes remain limited, and the fundamental relationship between temperature, structural evolution, and capacitive performance is still not fully understood.

Herein, corn straw-derived carbons (CS) were prepared at 600, 800, and 1000 °C through a simple additive-free carbonization route without chemical activation or external doping. Unlike previous studies primarily focused on maximizing capacitance through activation, templating, or heteroatom engineering, the present work aims to isolate the intrinsic influence of carbonization temperature on the structural evolution and electrochemical behavior of non-activated biomass-derived carbons. By systematically correlating temperature-induced changes in pore characteristics, surface chemistry, paramagnetic defect states, and structural ordering with electrochemical performance, this study provides improved understanding of temperature–structure–property relationships in additive-free biomass carbon systems. Although the electrochemical performance remains moderate compared with highly activated porous carbons, the findings provide mechanistic insight that may guide future activation and defect-engineering strategies toward sustainable carbon electrodes.

## 2. Materials and Methods

### 2.1. Materials

Corn straw was sourced from a farm in Harbin, Heilongjiang Province, China. 1-Methyl-2-pyrrolidinone (NMP), potassium hydroxide (KOH), and polyvinylidene difluoride (PVDF) were purchased from Sinopharm Chemical Reagent Co., Ltd. (Shanghai, China). Battery-grade conductive carbon black (Super P) was purchased from Shenzhen Kejing Zhida Technology Co., Ltd. (Shenzhen, China). Battery-grade nickel foam was purchased from Kunshan Jiayisheng Electronics Co., Ltd. (Kunshan, China). High-purity argon (Ar ≥ 99%) was purchased from Harbin Grand Gas Co., Ltd. (Harbin, China).

### 2.2. Synthesis of Carbon Materials

The raw corn straw was thoroughly washed with deionized (DI) water to eliminate adhered soil and surface contaminants, then dried at 100 °C for 12 h. The dried material was crushed and sieved through a 20-mesh sieve to ensure uniform particle size. The sieved biomass was placed in an alumina boat and maintained at 2 h at 600, 800, or 1000 °C with a rate of 5 °C min^−1^ under an argon atmosphere. Finally, the samples were naturally cooled to room temperature to obtain carbon without de-ashing.

De-ashing treatment: To remove residual inorganic components that may influence the structural and electrochemical behavior of biomass carbons, the carbonized products were subjected to a de-ashing treatment. The obtained carbons were washed with 10 wt% HCl under stirring at room temperature, followed by repeated rinsing with deionized water until a neutral pH. The washed samples were then dried at 80 °C. The final products were denoted CS-600, CS-800, and CS-1000, according to their respective carbonization temperatures.

Bulk mass (gravimetric) yield calculation method: Raw corn straw was dried at 100 °C for 12 h to constant weight, with five independent batches prepared at each temperature to ensure statistical reliability. For each batch, a known mass of dried precursor (*m_pre_*) was carbonized under identical conditions. After cooling under argon, the crude carbon was weighed to obtain *m_char_*_,*crude*_. The char was then subjected to acid washing, filtration, rinsing, and drying, after which the ash-free char mass (*m_char_*_,*ashed*_) was recorded. This procedure was performed for samples carbonized at 600, 800, and 1000 °C. The mass yield was calculated according to Equations (1)–(3) [24].

(1) $$\mathrm{Crude}\;\mathrm{char}\;\mathrm{yield}\;(\%)=\frac{m_{char,crude}}{m_{pre}}\times 100$$

(2) $$\mathrm{De}-\mathrm{ashed}\;\mathrm{char}\;\mathrm{yield}\;(\%)=\frac{m_{char,ashed}}{m_{pre}}\times 100$$

(3) $$\mathrm{Ash}\;\mathrm{removal}\;(\%)=\frac{m_{char,crude}-m_{char,ashed}}{m_{char,crude}}\times 100$$

### 2.3. Structure and Morphology Characterization

The crystal structure of the samples was acquired by X-ray diffraction (XRD; Malvern Panalytical Empyrean, Worcestershire, UK) with Cu Kα radiation (λ = 1.5406 Å) in the 2θ range of 5–90°. Raman spectroscopy (WITec Alpha 300 R, WITec GmbH, Ulm, Germany) with a laser wavelength of 532 nm was used to explore the ratio of I_D_/I_G_. The surface functional groups were determined using an FT-IR spectrometer (PerkinElmer, Waltham, MA, USA) in the transmission mode covering the range of 400–4000 cm^−1^. To verify the thermal stability of the carbon materials, thermal gravimetric analysis (TGA) was performed in the temperature range RT–750 °C using a NETZSCH instrument (Selb, Germany) operating in an air atmosphere at a heating rate of 5 °C min^−1^. The specific surface area and pore structure were analyzed using a Micromeritics ASAP 2460 surface area and porosity analyzer (Micromeritics Instrument Corporation, Norcross, GA, USA; Version 3.01.02). The pore size distribution was determined using the Barrett–Joyner–Halenda (BJH) model, and the SSA was measured using the Brunauer–Emmett–Teller (BET) method. The morphologies of the samples were examined using a scanning electron microscope (SEM, Hitachi-SU8020, Hitachi, Tokyo, Japan) and a transmission electron microscope (TEM, JEM-F200 (URP), JEOL, Tokyo, Japan). The oxygen vacancy concentration of the samples was recorded by electron paramagnetic resonance (EPR) spectra using a Bruker EMXplus-6/1 instrument (Bruker, Billerica, MA, USA). The elemental composition and chemical state of the materials were analyzed by X-ray photoemission spectroscopy (XPS, Thermo Scientific ESCALAB 250Xi, Thermo Scientific, Waltham, MA, USA). The binding energies were normalized considering the C 1s peak at 284.8 eV as a reference.

### 2.4. Electrochemical Measurements

Electrochemical tests were analyzed by the CHI760E workstation. The working electrode was fabricated by mixing carbon materials, PVDF, and Super P in a mass ratio of 8:1:1. Then, NMP was used as the solvent, and the mixture was stirred at 500 rpm for 12 h to form a slurry. Finally, the slurry was uniformly coated on the nickel foam substrate and dried at 60 °C for 12 h to obtain an electrode. The three-electrode system: Pt foil, Ag/AgCl, and as-prepared electrode served as the counter electrode, reference electrode, and working electrode, respectively. A volume of 1 M KOH is used as the electrolyte. Cyclic voltammetry (CV) tests were conducted at operating voltages of −1 V and 0 V at different scan rates. Galvanostatic charge/discharge (GCD) tests were conducted at different current densities. Electrochemical impedance spectroscopy (EIS) measurements were performed using a voltage amplitude of 5 mV of open-circuit potential between 10^−2^ and 10^5^ Hz.

In a three-electrode system, Equation (4) can determine the gravimetric specific capacitance (*C*, F g^−1^) of the as-prepared working electrode from the constant current charge/discharge curve [25].

(4) $$C_{g}=\frac{I\Delta t}{m\Delta V}$$

where *I* (A), Δ*t* (s), Δ*V* (V), and *m* (g) represent the discharge current, discharge time, potential window excluding IR drop, and mass of active material on the working electrode, respectively.

The symmetrical supercapacitor device was built with CS-800 as the positive and negative electrodes in a 1 M KOH aqueous electrolyte and separator to examine potential applications for energy storage. Furthermore, the assembled device was maintained at ambient temperature for over 12 h to ensure sufficient electrolyte infiltration. In the symmetric capacitor, Equation (5) was used to calculate the capacitance (*C*, F g^−1^) of the single electrode. Equations (6) and (7) were used to calculate the energy density (*E*, Wh kg^−1^) and power density (*P*, W kg^−1^), respectively [26].

(5) $$C=\frac{I\Delta t}{M\Delta V}$$

(6) $$E=\frac{C{\left(\Delta V\right)}^{2}}{2\times 3.6}$$

(7) $$P=\frac{3600E}{\Delta t}$$

where *M* (g) represents the total mass of the active materials in the two electrodes.

## 3. Results and Discussion

### 3.1. Morphology and Pore Structure Analysis

**Figure 1:**
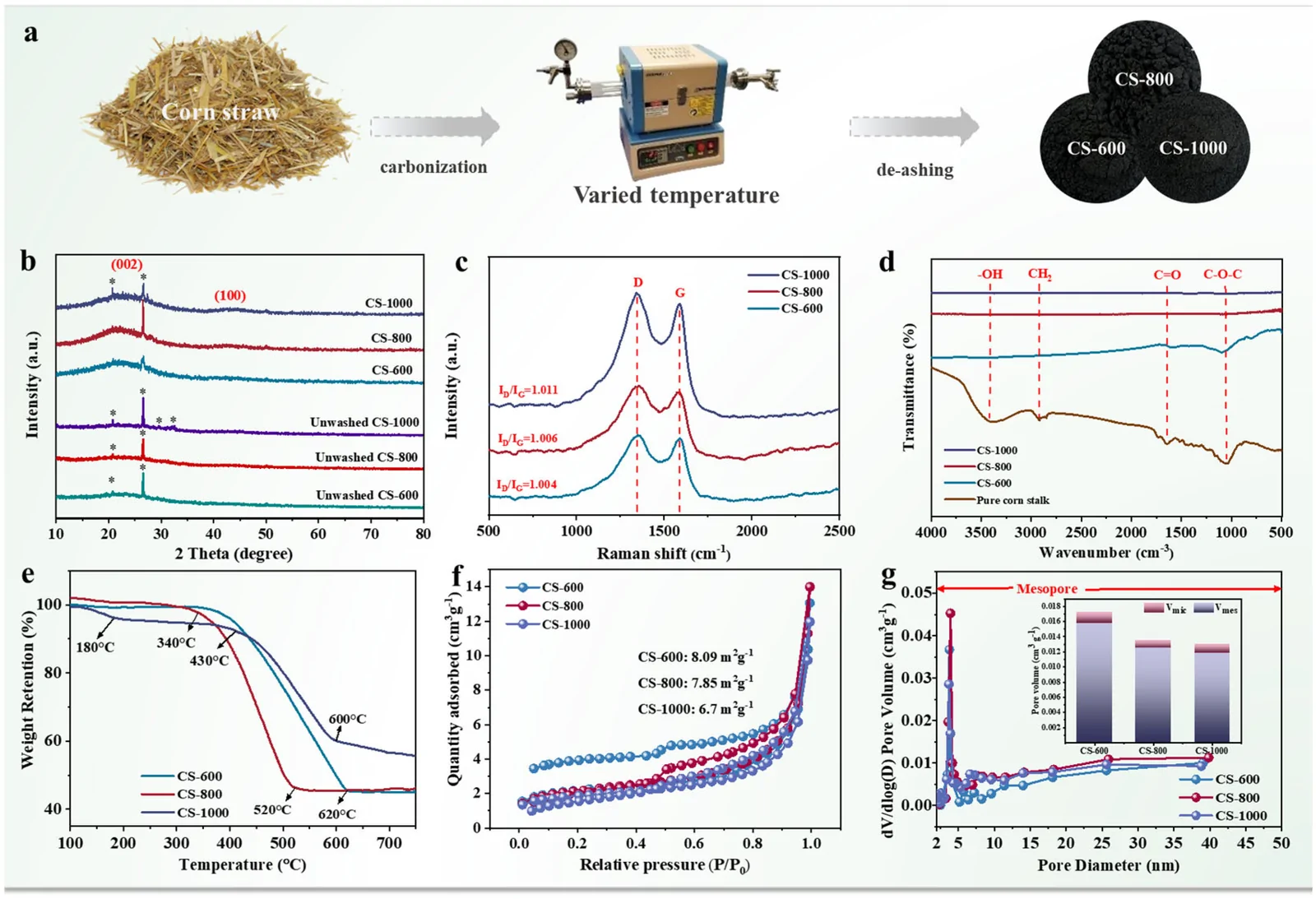
The analysis of the structure and pore distribution of the CS-X samples. (**a**) Schematic diagram illustrating the preparation process, (**b**) XRD patterns, (**c**) Raman spectrum, (**d**) FT-IR spectra, (**e**) TGA curves, (**f**) N_2_ adsorption–desorption isotherm, and (**g**) pore size distribution (inset: pore volume histogram) of the CS-X samples. The peaks marked with an asterisk (*) in the XRD patterns correspond to crystalline SiO_2_ (quartz), indicating residual inorganic ash-derived impurities in the unwashed samples.

Corn straw-derived carbons were prepared via direct carbonization followed by de-ashing as illustrated in Figure 1a. The activated agent-free strategy can isolate the intrinsic effects of carbonization temperature on the evolution of the carbon framework, surface chemistry, and electrochemical behavior. During carbonization, the gradual thermal decomposition of the biomass matrix leads to the release of volatile species such as CO_2_, CO, H_2_O, and light hydrocarbons, and is accompanied by the gradual aromatization and condensation of the carbon framework [27,28,29]. The XRD patterns before and after acid washing of the CS-X materials (Figure 1b) exhibited a broad diffraction feature centered at ~22–25°, characteristic of turbostratically disordered (002) carbon, along with a weak and broadened reflection near ~43° corresponding to the (100) plane [30]. Superimposed on this background, sharp reflections at ~20.7° and ~26.6° are observed and are assigned to crystalline SiO_2_ (quartz), marked with *, suggesting the presence of inorganic impurities (SiO_x_ or ash). These peaks are significantly more intense in the unwashed samples, indicating a higher content of mineral residues originating from the biomass precursor. The presence of such impurities is commonly associated with the intrinsic ash content of the biomass and can be further influenced by the carbonization process [31]. After acid washing, the intensity of these SiO_2_-related peaks decreases markedly, confirming the effective removal of acid-soluble inorganic species. However, weak residual peaks are still observed, which can be attributed to the acid-insoluble silica inherently present in the biomass structure [32]. TEM and SAED analysis further confirm that the carbon matrix remains predominantly amorphous/turbostratic, indicating that these sharp peaks do not originate from crystalline carbon but from residual inorganic phases. Two characteristic Raman bands centered at ~1358 cm^−1^ (D band) and ~1592 cm^−1^ (G band) were observed for all CS-X materials (Figure 1c). The Raman peaks exhibited negligible variations, suggesting that the overall structural disorder and graphitic characteristics remain broadly comparable within the investigated temperature range.

The FT-IR spectra of CS-X (Figure 1d) shows that the content of functional groups on the surface of the materials varies significantly at different pyrolysis temperatures. However, at pyrolysis temperatures above 600 °C, most of the absorption peaks become very weak and their intensity continues to decrease, indicating a serious decomposition of the functional groups on the surface of the biochar. As the carbonization temperature increases, the stretching vibration peak of -OH at a wavelength of 3408 cm^−1^ gradually weakens, which may be due to the detachment of bound water during pyrolysis, leading to the breaking of hydroxyl groups bound by hydrogen bonds [33]. The stretching vibration peak of aliphatic CH_2_ around 2920 cm^−1^ shows no significant change in peak intensity with increasing temperature. The CS-600 sample exhibits noticeable absorption bands at approximately 1096 cm^−1^ and 1637 cm^−1^, which can be attributed to C–O–C, and C=O stretching vibrations, respectively, originating from the residual oxygen-containing groups of the biomass precursor [21].

As shown in Figure 1e, thermogravimetric analysis (TGA) was carried out in air from 100 °C to 750 °C to evaluate the thermal stability and inorganic residue content of the CS-X samples. All samples exhibit two distinct stages of weight loss. The first stage is associated with the physical adsorption of moisture, and the second stage is associated with the oxidative decomposition of biomass-derived carbonaceous components [34]. The total mass losses of CS-600, CS-800, and CS-1000 are approximately 55%, 54%, and 45%, respectively. The decrease in char yield and the concurrent increase in TGA residual mass with increasing carbonization temperature indicate a transition from mass retention to structural stabilization, whereby fewer but more thermally robust carbon domains are formed [35,36]. It should be noted that char yield is referenced to the initial raw corn straw mass, whereas TGA residue is referenced to the pre-carbonized sample; therefore, the two parameters reflect different aspects of the carbonization process. Furthermore, bulk mass yield calculations were performed based on gravimetric measurements. As the carbonization temperature increased from 600 to 1000 °C, the gravimetric yield of the carbon products gradually decreased, reflecting enhanced thermal decomposition and volatilization of the biomass precursor. The crude char yields for CS-600, CS-800, and CS-1000 were 29.2 ± 0.8%, 28.5 ± 0.6%, and 27.5 ± 0.4%, respectively. After acid washing, the corresponding de-ashed char yields decreased to 22.1 ± 0.7%, 21.4 ± 0.5%, and 19.4 ± 0.3%, indicating an ash removal efficiency of approximately 24.5–29.5 wt%.

Nitrogen adsorption–desorption measurements were conducted to investigate the pore structures of the CS-X samples. As illustrated in Figure 1f, the non-closed isotherm of CS-600 can be reasonably ascribed to the high density of oxygen-containing surface functional groups, which results in adsorption irreversibility. Conversely, the non-closure of the CS-1000 isotherm is more likely caused by pore structure collapse or contraction induced by excessive carbonization and framework shrinkage at elevated temperatures. The specific surface areas (SSA) of CS-600, CS-800, and CS-1000 are 8.09, 7.85, and 6.70 m^2^ g^−1^, respectively. The monotonic decrease in SSA with increasing carbonization temperature is attributed to structural densification and the partial collapse of thermolabile pore frameworks during high-temperature treatment [37]. Figure 1g illustrates the pore size distribution obtained from the adsorption branch utilizing a BJH-type model. All samples exhibit a predominant mesopore distribution between roughly 2 and 40 nm, with a limited but measurable micropore contribution (<2 nm). This verifies that the carbon’s porosity is predominantly mesoporous, aligning with the isotherm forms depicted in Figure 1f. Moreover, excessively small pores may increase the total surface area while limiting ion transport [38]. The relationship between specific surface area and capacitance is not directly proportional [39]. Therefore, pore accessibility may strongly influence electrochemical behavior. Although the BET surface area remained relatively low, the electrochemical response may depend more strongly on effective surface area and pore accessibility rather than total surface area alone.

To further elucidate the pore characteristics of the carbon materials, the accumulated surface area was analyzed as a function of pore width, as shown in Figure S1, where the sharp increase at small pore widths indicates that a substantial fraction of the surface area originates from micro- and small mesopores (around 5–6 nm). Furthermore, to obtain a more comprehensive understanding of the pore characteristics, additional micropore analyses were performed using the t-plot method and the NLDFT model. The t-plot results (Table 1) indicate that the CS-X samples possess relatively small micropore surface areas (2.74–3.63 m^2^ g^−1^) and micropore volumes on the order of ~10^−3^ cm^3^ g^−1^, suggesting limited microporous development within the carbon framework. The external surface areas derived from the t-plot analysis are comparable to the measured BET surface areas, indicating that the overall porosity of the materials is relatively low.

The NLDFT pore size distribution (Figure S2) further reveals that only a minor fraction of pores exists in the micropore region (<2 nm), while the primary pore contribution appears in the small-mesopore range (~5–10 nm). These observations are consistent with the low BET surface areas measured for the CS-X materials. To further evaluate surface wettability, time-dependent deionized-water contact-angle measurements were performed (Figure S3). All samples exhibited progressively decreasing contact angles with time, indicating gradual wetting behavior. Among them, CS-800 showed relatively lower contact angles and faster wetting behavior, suggesting improved electrolyte accessibility. Enhanced wettability may facilitate ion penetration and utilization of accessible active sites, which could partially contribute to the improved electrochemical response despite the similarly low BET surface areas. Although the BET surface areas of the CS-X samples are similarly low, noticeable differences in capacitance were still observed, suggesting that electrochemical behavior is influenced not solely by total surface area, but also by electrolyte accessibility and surface-related structural characteristics.

**Figure 2:**
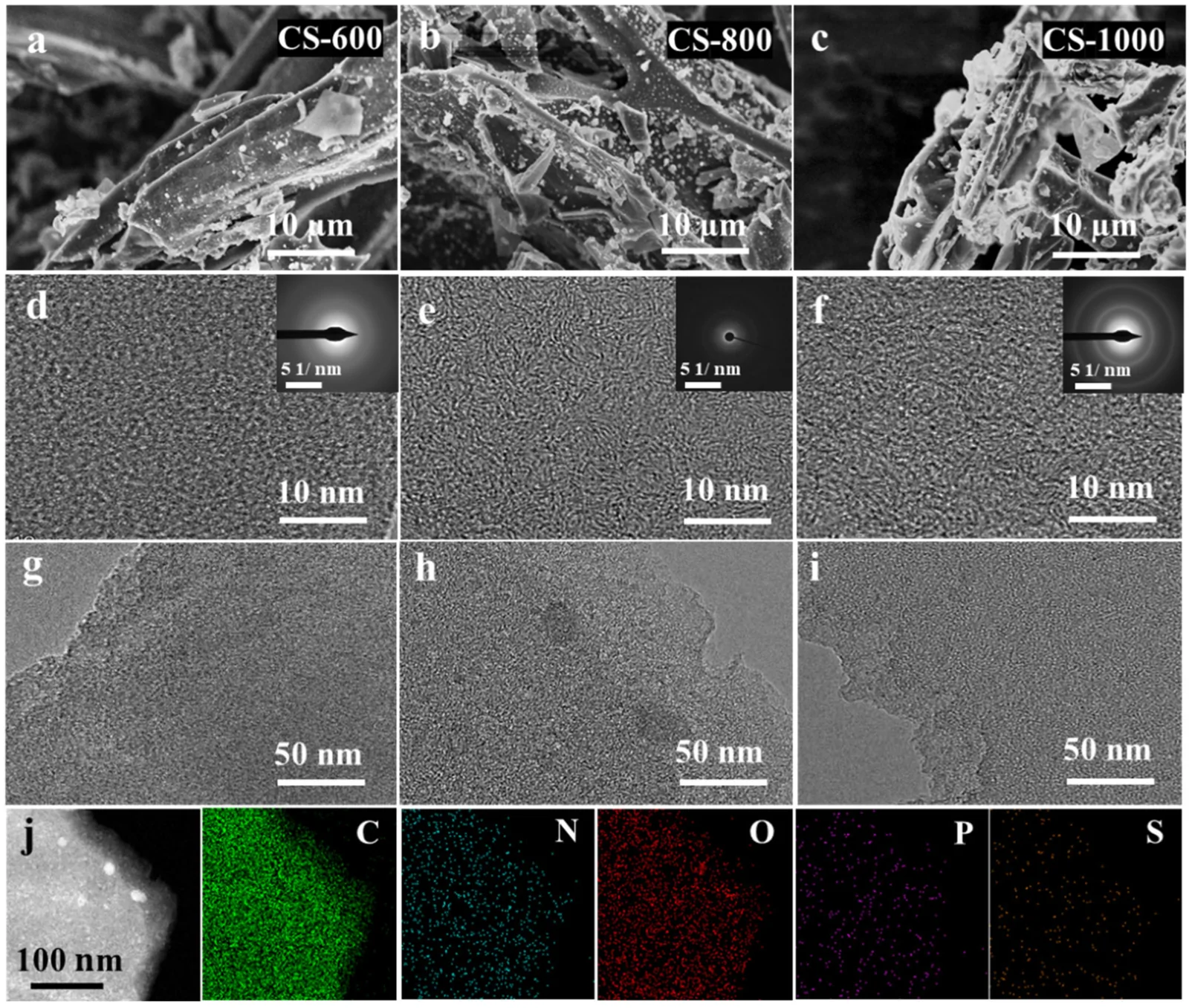
The morphology and structural characterization of CS-X samples: SEM images of (**a**) CS-600, (**b**) CS-800, and (**c**) CS-1000; TEM images and corresponding SAED patterns of (**d**,**g**) CS-600, (**e**,**h**) CS-800, and (**f**,**i**) CS-1000; and (**j**) EDS elemental mapping of CS-800.

Moreover, the SEM images (Figure 2a–c) reveal that all CS-X samples retain the inherent morphology of the corn straw precursor after carbonization. The CS-600 sample exhibits a relatively smooth and compact surface with limited pore development. With increasing carbonization temperature, the surface becomes progressively rougher and more fragmented. Notably, CS-800 shows a more developed porous texture with abundant surface irregularities, indicating partial decomposition of volatile components and pore formation. In contrast, CS-1000 displays a collapsed and fractured structure, suggesting excessive carbonization leading to structural shrinkage and pore coalescence. The TEM images at the 10 nm scale (Figure 2d–f) demonstrate that all samples consist of predominantly amorphous carbon structures without obvious long-range ordered graphitic lattices. However, subtle differences can be observed with increasing temperature. CS-600 shows relatively dense and disordered carbon domains, while CS-800 exhibits a more open and loosely packed nanostructure, which is favorable for ion transport and electrolyte accessibility. For CS-1000, the structure appears more compact again, likely due to pore collapse and carbon framework densification at higher temperatures. At a larger scale (50 nm, Figure 2g–i), CS-800 presents a more homogeneous and interconnected porous network compared to CS-600 and CS-1000, further supporting comparatively more developed microstructural characteristics within the present carbonization series. This suggests that high-temperature carbonization can form a carbon skeleton, but the insufficient pore development limits the surface area [40]. In contrast, low-temperature carbonization still results in insufficient porosity, reflecting limitations in regulating the pore structure [41]. Moreover, excessively small pores may increase the total surface area while limiting ion transport [38]. Therefore, pore accessibility may strongly influence electrochemical behavior.

The corresponding SAED patterns (insets in Figure 2d–f) display diffuse halo rings rather than sharp diffraction spots, indicating that all samples predominantly exhibit amorphous characteristics. The absence of well-defined crystalline diffraction rings suggests only limited local structural ordering, consistent with the behavior typically reported for biomass-derived carbons prepared without activation or catalytic graphitization strategies. This observation corroborates the XRD results, confirming that the broad (002) reflection arises from disordered carbon stacking, while the sharp diffraction peaks observed in the XRD patterns do not originate from crystalline carbon but are instead associated with trace inorganic residues. Furthermore, EDS elemental mapping of CS-800 (Figure 2j and Figure S4) confirms a homogeneous distribution of C, N, O, P, and S throughout the carbon matrix, demonstrating successful in situ self-doping during carbonization, consistent with previous reports [42,43].

**Figure 3:**
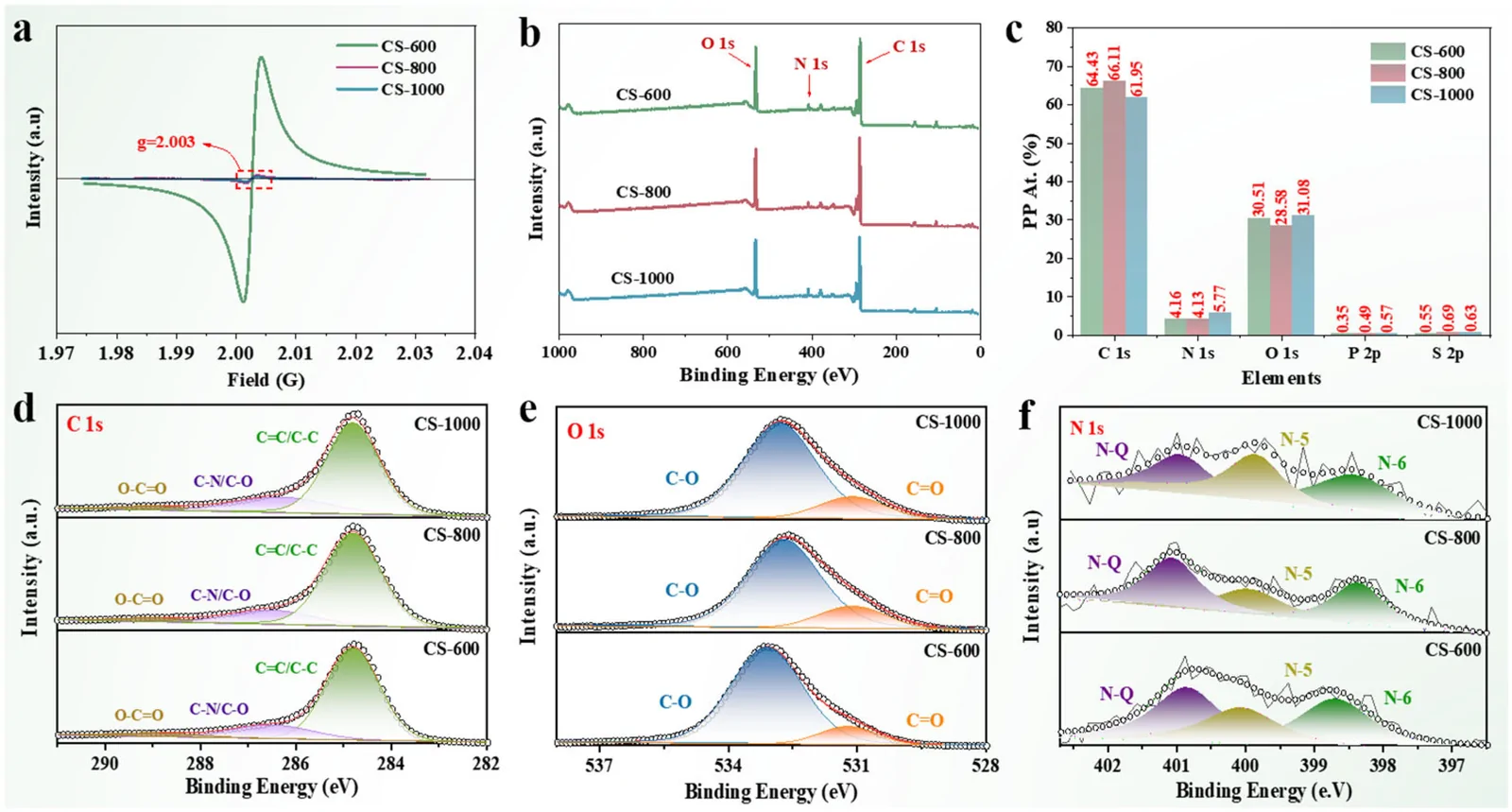
The element analysis of CS-X samples. (**a**) EPR plot, (**b**) XPS survey spectra, (**c**) elemental distribution, and high-resolution XPS spectra of C 1s (**d**), O 1s (**e**), and N 1s (**f**) of as-prepared materials.

As shown in Figure 3a, the EPR spectra of CS-600, CS-800, and CS-1000 reveal the presence of unpaired electrons in all samples, with a characteristic signal at g = 2.003. These signals are commonly associated with carbon-centered paramagnetic defects and oxygen-containing surface groups, while the electrochemical behavior does not directly correlate with EPR intensity alone [44,45,46]. Among the samples, CS-800 exhibits an intermediate EPR intensity, whereas the reduced EPR signal observed for CS-1000 may be associated with partial defect healing and slight local structural ordering of the carbon framework at elevated carbonization temperatures. In contrast, the comparatively higher EPR intensity of CS-600 suggests a greater abundance of defect-related paramagnetic states and surface functionalities. However, the electrochemical performance trend does not directly follow the EPR intensity, indicating that defect-related features alone do not solely determine the electrochemical response. Instead, electrochemical behavior is likely influenced collectively by pore accessibility, surface wettability, and resistance behavior.

XPS analysis (Figure 3b–f) confirms the presence of C, O, N, P, and S in all CS-X samples. Increasing carbonization temperature to 800 °C increases the carbon content while reducing oxygen concentration. The high-resolution C 1s spectra reveal dominant *sp*^2^/*sp*^3^-hybridized carbon (C–C/C=C, ~284.8 eV), along with an oxygen-containing functional group attributed to C–O/C–N (~286.5 eV) and carboxyl groups (O–C=O, ~288.9 eV), consistent with recent carbon material studies using XPS. Correspondingly, the O 1s spectra exhibit peaks at ~531.1 eV and ~532.8 eV, which are assigned to carbonyl oxygen (C=O) and hydroxyl/ether oxygen (C–O), respectively [47,48,49]. Likewise, the N 1s spectra reveal three peaks with the binding energies correlated to pyrrolic-N (N-5), quaternary-N (N-Q), and pyridinic-N (N-6). Pyridine nitrogen provides additional electron and charge mobility to enhance electrical conductivity and provides active sites for redox reactions that may contribute to interfacial charge-transfer behavior and surface-related electrochemical activity. Pyrrole nitrogen improves interfacial and cyclic performance, while graphitic nitrogen provides additional sites for electron transfer [50,51]. The retention of nitrogen functional groups in the derived carbon without the need for additional activation or doping suggests that this approach allows for self-doping through the intrinsic composition of the raw material. These results suggest that corn straw-derived carbon may serve as a sustainable precursor for carbon-based electrochemical energy-storage materials.

Raman and XPS analyses were performed to evaluate the structural characteristics and surface chemistry of the CS-X carbon materials (Table S1). Among the samples, CS-800 exhibits a balanced distribution of carbon bonding configurations together with moderate oxygen content, which may improve surface wettability and facilitate electrolyte ion accessibility at the electrode–electrolyte interface. These structural characteristics, together with the comparatively favorable pore accessibility and resistance behavior, are consistent with the relatively improved electrochemical performance observed for CS-800.

### 3.2. Electrochemical Performance in a Three-Electrode System

The electrochemical performance of CS-600, CS-800, and CS-1000 was evaluated in a 1 M KOH electrolyte using a three-electrode configuration. To determine the suitable operating voltage range for the electrochemical measurement, the CS-800 electrode was tested at various voltage ranges. Figure S5a shows the typical cyclic voltammetry (CV) curves at 5 mV s^−1^ from −0.6~−1.2 V to 0, respectively, and curves maintain a quasi-rectangular-like shape at 1 V. Thus, the operating voltage range was set between −1 and 0 V. Furthermore, the EDLC behavior was evaluated within the selected voltage window by recording CV curves at scan rates ranging from 100 to 800 mV s^−1^ and conducting GCD measurements at current densities between 0.5 and 11 A g^−1^, as shown in Figure S5b and S5c, respectively.

**Figure 4:**
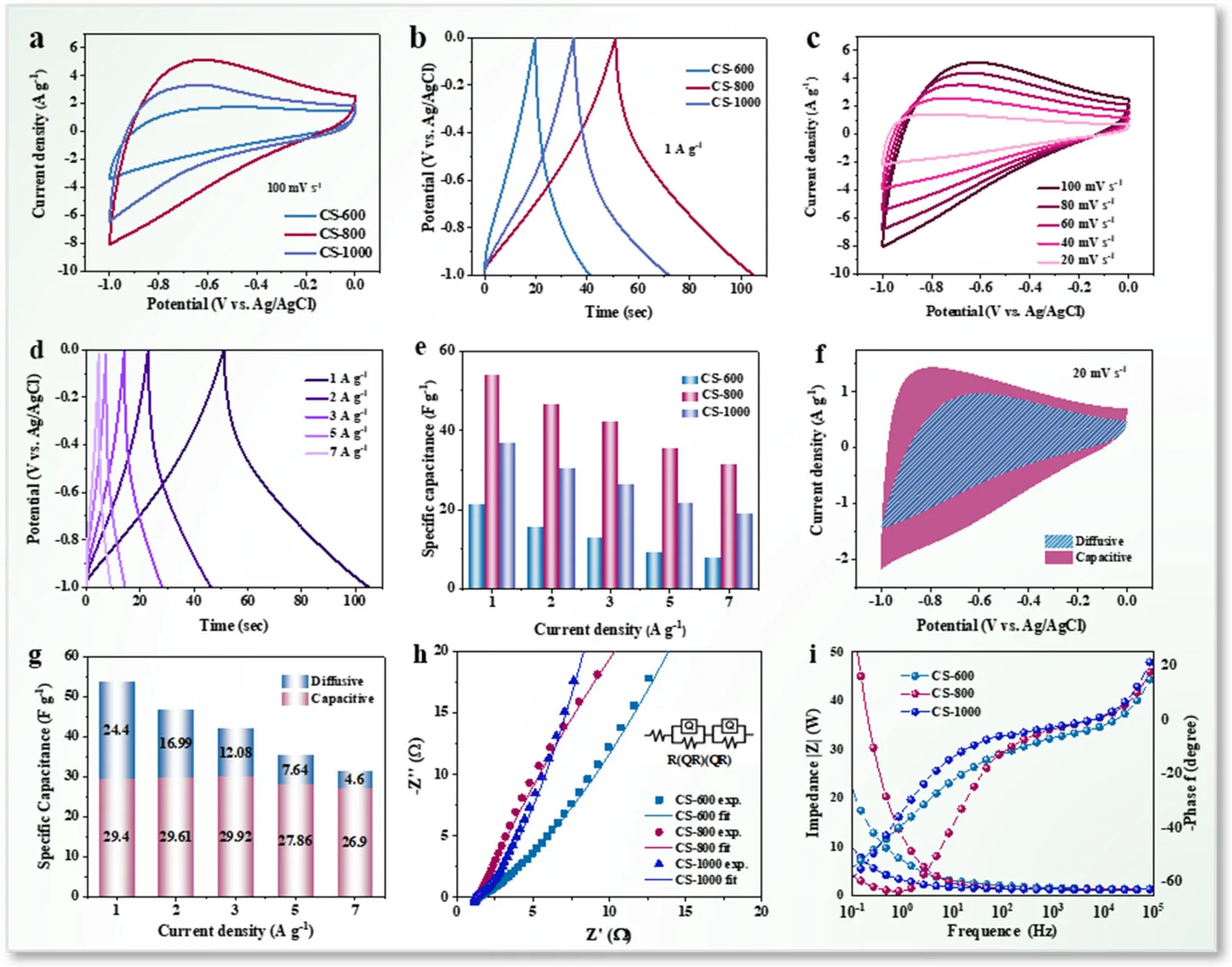
Electrochemical performances of CS-X electrodes: (**a**) CV curves at a scan rate of 100 mV s^−1^, (**b**) GCD curves at a current density of 1 A g^−1^, (**c**) CV curves at various scan rates of the CS-800, (**d**) GCD curves at different current densities of the CS-800, (**e**) specific capacitance at various current densities, (**f**) voltammograms deconvoluted into capacitive and diffusion-controlled contributions scanned at 20 mV s^−1^, (**g**) specific-capacitance values separated into capacitive and diffusive components at various current densities (1–7 A g^−1^), (**h**) Nyquist plots together with fitted curves based on the R(QR)(QR) equivalent circuit model, and (**i**) Bode graph.

Figure 4a presents the cyclic voltammetry (CV) curves recorded at a scan rate of 100 mV s^−1^ within the potential window of −1.0 to 0 V. All samples exhibit quasi-rectangular CV profiles, indicating typical electric double-layer capacitance (EDLC) behavior with additional pseudocapacitive contributions. The latter arise from surface defects and oxygen-containing functional groups, such as hydroxyl, carboxyl, and phenolic species, which facilitate reversible Faradaic reactions at the electrode/electrolyte interface [52]. Among the three samples, CS-800 demonstrates the highest current response, suggesting superior charge-storage capability. The galvanostatic charge–discharge (GCD) curves shown in Figure 4b further corroborate the CV results. CS-800 exhibits the longest discharge time under identical current conditions, reflecting its higher capacitance. Based on the GCD analysis, the specific capacitances of CS-600, CS-800, and CS-1000 are 21.2, 53.8, and 36.7 F g^−1^ at 1 A g^−1^, respectively. The significantly enhanced capacitance of CS-800 is attributed to the comparatively balanced structural characteristics, including in the defect density, pore characteristics, and carbon microstructure. Although CS-600 exhibited a slightly larger specific surface area, its lower carbonization temperature may have resulted in less developed conductive pathways and structural characteristics. In contrast, CS-1000 may exhibit improved electron transport associated with slight structural ordering; however, elevated carbonization temperature could also contribute to structural densification and reduced accessible surface characteristics. Among the investigated samples, CS-800 offers the most favorable combination of moderate graphitization, abundant defects, and mesoporous architecture, which is consistent with the improved electrochemical response. These results confirm that specific capacitance is not determined solely by surface area; rather, it is governed by the coupled effects of pore size distribution, defect concentration, and structural ordering, all of which are modulated by the carbonization temperature.

Figure 4c shows the CV curves of CS-800 recorded at scan rates from 20 to 100 mV s^−1^. All curves retain a quasi-rectangular shape without noticeable distortion, indicating that CS-800 maintains rapid charge–discharge kinetics and robust capacitive behavior even at high scan rates. Compared with CS-600 (Figure S6a) and CS-1000 (Figure S7a), CS-800 exhibits a significantly higher current response across all scan rates, highlighting its superior electrochemical activity. The slight deviation from an ideal rectangular profile is attributed to pseudocapacitive contributions superimposed on the dominant EDLC mechanism. The GCD curves (Figure 4d) display symmetric triangular shapes without discernible potential plateaus, even when the current density increases sevenfold from 1 to 7 A g^−1^. This confirms the fast and reversible charge-storage process of CS-800. As shown in Figure 4e, the CS-800 electrode delivers a specific capacitance of 31.5 F g^−1^ at 7 A g^−1^, corresponding to a capacitance retention of 58.55% relative to its value at 1 A g^−1^. This performance is substantially higher than those of CS-600 (7.7 F g^−1^, Figure S6b) and CS-1000 (18.9 F g^−1^, Figure S7b). The decline in capacitance at higher current densities observed for all CS-X samples is attributed to the limited diffusion of electrolyte ions into the internal pore structure under fast charge–discharge conditions.

To evaluate the role of porosity in charge storage, the specific capacitance was correlated with the pore structure parameters (Table 1). Although CS-800 exhibits the highest capacitance and a slightly higher total pore volume, the variation in pore volume among the samples is relatively small. Moreover, no clear correlation is observed between micropore volume and capacitance. These results suggest that porosity does not play a dominant role in determining capacitance differences. Previous studies have demonstrated that biomass-derived carbons obtained via direct carbonization without activation typically exhibit relatively low capacitance due to limited surface area and poor pore accessibility. For instance, capacitance values as low as ~24 F g^−1^ have been reported for such materials, whereas moderate values on the order of ~80 F g^−1^ are also observed depending on the structural properties [53,54,55]. These results confirm that the performance reported in this work falls within the expected range for non-activated biomass-derived carbons. Importantly, the present study focuses on elucidating the structure–property relationship governed by carbonization temperature, rather than maximizing capacitance through chemical activation.

Figure 4f presents the capacitive and diffusion-controlled contributions extracted from the CV curves at 20 mV s^−1^, while additional deconvoluted profiles at 40 and 100 mV s^−1^ are shown in Figure S8. Figure 4g summarizes the separated surface-controlled and diffusion-controlled capacitance contributions at different current densities. The diffusion-controlled contribution gradually decreases with increasing current density because electrolyte ions have insufficient time to access less accessible pores under fast charge–discharge conditions. In contrast, the surface-controlled contribution becomes comparatively more dominant at higher rates due to rapid charge storage at accessible surface sites. These observations suggest that charge storage in CS-800 is governed predominantly by surface-controlled capacitive behavior, while diffusion-limited processes become progressively restricted at high current densities. Figure 4h presents the Nyquist plots of the CS-X electrodes together with the fitted curves based on the R(QR)(QR) equivalent circuit model. The fitted results show good agreement with the experimental data, indicating reliable fitting quality. The series resistances (Rs ≈ 137, 123, and 116 mΩ for CS-600, CS-800, and CS-1000, respectively) suggest relatively low internal resistance and limited IR drop during operation. In addition, the comparatively small charge-transfer resistance and steeper low-frequency response indicate relatively improved interfacial ion transport and charge-storage behavior. The corresponding 2D Bode analysis (Figure 4i) provides further insight into the frequency-dependent response and rate-limiting processes across the measured frequency range. Among the samples, CS-800 exhibits a comparatively more favorable frequency response, which is consistent with its improved electrochemical performance and ion transport characteristics relative to CS-600 and CS-1000.

To further clarify the relationship between electrical double-layer capacitance and the specific surface area of each material, electrochemical analyses were performed in conjunction with structural characterization. Figure S9a–c shows the CV curves of CS-600, CS-800, and CS-1000 at different scan rates. The curves exhibit quasi-rectangular shapes, indicating typical EDLC behavior. To further investigate the charge-storage mechanism, the relationship between current and scan rate was analyzed according to $i=av^{b}$ and the corresponding b-values were determined (Figure S9d). The obtained b-values range from 0.60 to 0.78, indicating that the charge-storage process is mainly surface-controlled with some contribution from ion diffusion. Notably, although EDLC is commonly correlated with specific surface area, the BET values of the three samples are similar and relatively low, while their capacitance differs significantly. This suggests that the capacitance is not directly proportional to the total surface area, but is more strongly influenced by the accessibility of active sites and ion transport within the porous structure.

### 3.3. Energy-Storage Performance of the Symmetric Supercapacitor

**Figure 5:**
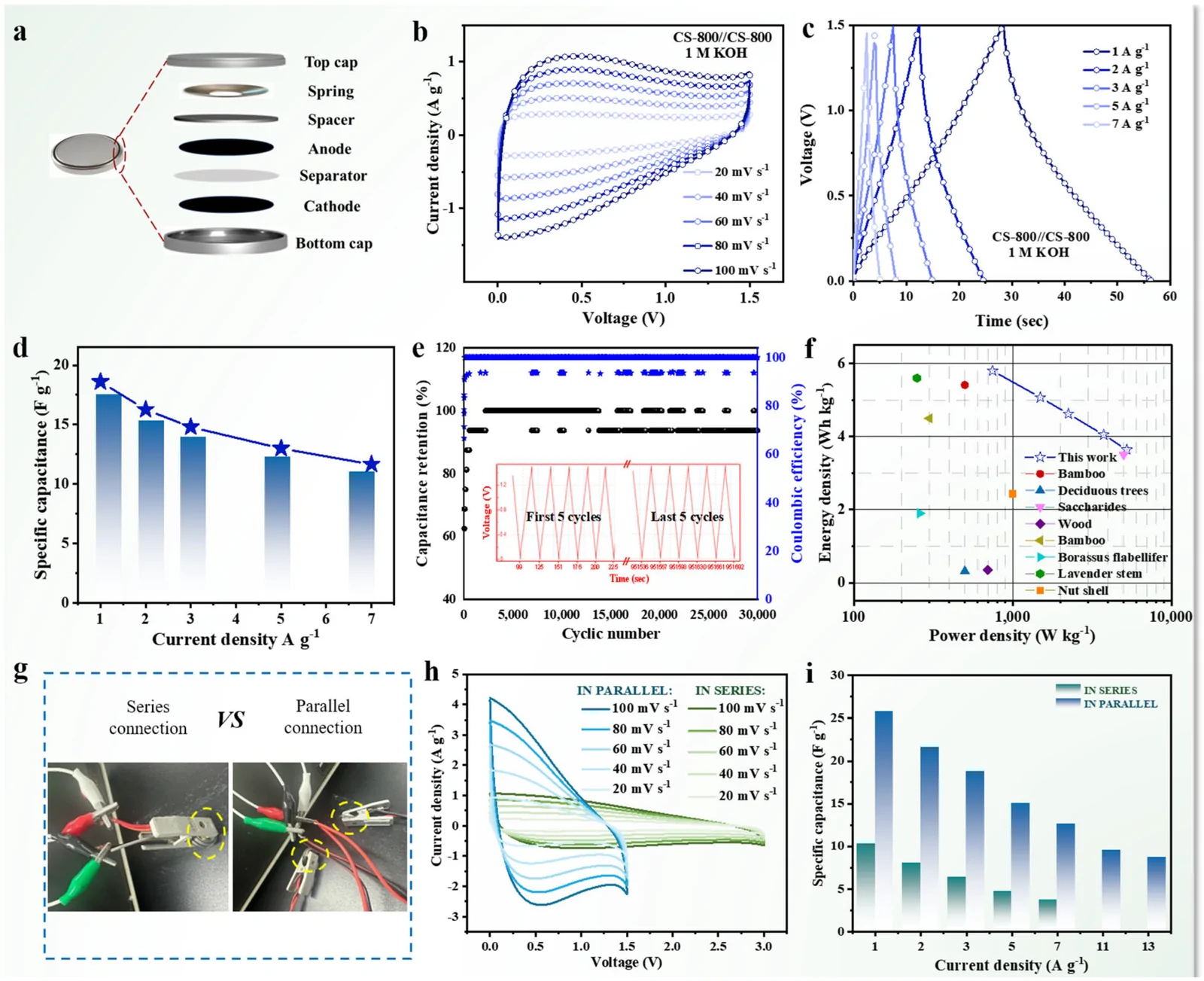
Electrochemical performances of CS-800//CS-800 SSC: (**a**) Schematic diagram of the SSC device, (**b**) CV curves at various scan rates, (**c**) GCD curves at different current densities, (**d**) rate performance, (**e**) cycling stability and Coulombic efficiency measured over 30,000 charge–discharge cycles at 2 A g^−1^, with inset showing the first five and last five GCD profiles, (**f**) Ragone plots, (**g**) device configuration and electrical connections, (**h**) CV curves of parallel vs. series connections, and (**i**) specific-capacitance comparison.

The electrochemical performance of CS-800 was further evaluated in a symmetric supercapacitor (CS-800//CS-800 SSC), using 1 M KOH as the electrolyte and glass fiber as the separator (Figure 5a). The CV curves recorded at various scan rates (Figure 5b) exhibit characteristic quasi-rectangular shapes without significant distortion, indicating relatively efficient ion transport and capacitive behavior. Correspondingly, the GCD curves at multiple current densities (Figure 5c) present nearly symmetric triangular profiles, confirming the good rate capability and reversible charge storage of the device. As shown in Figure 5d, the CS-800//CS-800 SSC delivers specific capacitances of 18.6, 16.3, 14.8, 13.0, and 11.7 F g^−1^ at current densities of 1, 2, 3, 5, and 7 A g^−1^, respectively. The gradual decline in capacitance at higher current densities reflects the expected limitations in ion diffusion within the electrode structure during rapid charge–discharge processes. Overall, the results suggest relatively stable capacitive behavior and favorable rate performance rather than high-performance energy-storage characteristics.

The CS-800//CS-800 symmetric device demonstrated stable cycling performance, maintaining capacitance retention of approximately 93.75% after 30,000 charge–discharge cycles at 2 A g^−1^ (Figure 5e). In addition, the Coulombic efficiency remained close to 100% throughout cycling, indicating highly reversible charge-storage behavior and relatively stable electrochemical operation within the applied 1.5 V voltage window. The inset displays the first five and last five galvanostatic charge–discharge profiles, which exhibit largely overlapping characteristics, suggesting limited degradation and preserved electrochemical reversibility during prolonged cycling. Coulombic efficiency, IR drop, and equivalent series resistance (ESR) values were calculated and summarized in Table 2. The Coulombic efficiency remained above 92%, indicating relatively reversible charge-storage behavior. ESR values were estimated from the IR drop using $ESR=\Delta V_{IR}/2I$. These results further support the relatively stable electrochemical behavior of the CS-800//CS-800 symmetric device.

Ragone plots of energy density versus power density indicate that CS-800 retains comparatively favorable energy density at elevated power densities, suggesting good rate capability (Figure 5f). Furthermore, as summarized in Table 3, activated and structurally engineered biomass-derived carbons generally exhibit larger surface areas and higher capacitance values, whereas directly carbonized or additive-free systems typically show more moderate electrochemical performance. Despite the similarly low BET surface areas of the present CS-X samples, noticeable differences in capacitance were still observed, suggesting that charge-storage behavior is not governed solely by surface area. These findings provide insight into temperature-dependent structure–property relationships in additive-free biomass-derived carbons.

To evaluate the practical applications, two identical CS-800//CS-800 SSC cells were connected in parallel and series topologies, respectively (Figure 5g). The CV curves (Figure 5h) show that the voltage range of the series configuration is almost twice that of a single cell, while the parallel configuration increases the effective capacitance, consistent with the theoretical principles of capacitors. Figure 5i compares the specific capacitance of the two setups. While the parallel assembly provides higher capacitance at a given operating voltage, the series connection is advantageous for applications requiring an expanded voltage window, highlighting the intrinsic trade-off between capacitance and operating voltage in device integration.

## 4. Conclusions

In summary, porous carbons were successfully derived from corn straw at different carbonization temperatures, resulting in variations in the defect-related characteristics, pore features, specific surface areas, and local structural ordering. These structural variations collectively influence the electrochemical behavior, suggesting that charge-storage performance is not governed solely by surface area or porosity but may also be affected by surface functionalities and other intrinsic structural characteristics. Among the samples, CS-800 exhibits the highest specific capacitance of 53.8 F g^−1^ at 1 A g^−1^, reflecting comparatively balanced structural characteristics, including defect density, pore characteristics, and carbon microstructure. The symmetric CS-800//CS-800 supercapacitor demonstrated stable cycling stability, favorable rate capability, and a maximum energy density of 5.8 Wh kg^−1^ at a power density of 750 W kg^−1^ in 1 M KOH. Overall, this study provides insight into the influence of carbonization temperature on the structural evolution and electrochemical behavior of additive-free biomass-derived carbons, establishing a useful basis for the future optimization and development of sustainable carbon-based electrodes for electrochemical energy-storage applications.

## Figures and Tables

**Table 1:** Pore structure parameters of CS-600, CS-800, and CS-1000 derived from N_2_ adsorption–desorption analysis.

Sample	BET Surface Area (m^2^ g^−1^)	T-Plot Micropore Area (m^2^ g^−1^)	Total Pore Volume (cm^3^ g^−1^)	T-Plot Micropore Volume (cm^3^ g^−1^)
CS-600	8.0934	3.6257	0.0202	0.0014
CS-800	7.8495	2.7431	0.0216	0.0011
CS-1000	6.6534	2.8189	0.0185	0.0012

**Table 2:** Coulombic efficiency, IR drop, and apparent ESR values of the CS-800//CS-800 symmetric supercapacitor at different current densities.

Current Density (A g^−1^)	Charging Time (s)	Discharging Time (s)	Coulombic Efficiency (%)	IR Drop, ΔV_IR_ (V)	Applied Current (A)	Apparent ESR from IR Drop (Ω)
1	28.4	27.9	98.2	0.018	0.0012	7.5
2	12.4	12.2	98.4	0.040	0.0024	8.3
3	7.5	7.4	98.7	0.068	0.0036	9.4
5	4.1	3.9	95.1	0.073	0.0060	6.1
7	2.5	2.3	92.0	0.117	0.0084	7.0

**Table 3:** Comparison of preparation methods and electrochemical properties of activated corn-derived carbon electrodes and directly carbonized biomass-derived carbons reported for supercapacitor applications.

Material	Method	Surface Area (m^2^ g^−1^)	Capacitance (F g^−1^)/Current Density (A g^−1^) (Electrolyte)	Voltage Window (V)	Cycling Stability (Cycle)	Ref.
A. Activated/engineered corn-derived systems						
Corn straw	ZnCl_2_ N-doped	1123	321/0.5 (6 M KOH)	N/A	93% (10,000)	[56]
Corn stem	KOH activation	1420	232/1 (6 M KOH)	N/A	91% (10,000)	[57]
Corn straw	KOH activation	2667	192/0.5 (1 M H_2_SO_4_)	0–1	94.1% (10,000)	[58]
			258/0.5 (1 M Et_4_NBF_4_)	0–2.5	80% (10,000)	
Corn stover	KOH activation	1607	310/0.02 (KOH)	0–1	95% (100)	[59]
Corn stover	Hydrothermal carbonization	215	242/0.05 (2 M KOH)	N/A	92% (2500)	[60]
Corn straw	N- and P-co-doped	1905	379/0.5 (6 M KOH)	N/A	98% (10,000)	[61]
Corn straw	KOH activation/zinc acetate template	1395	254/0.5 (6 M KOH)	N/A	97% (15,000)	[62]
Corn straw	Microwave-assisted hydrothermal carbonization	1781	98/0.5 (1 M TEA BF_4_-PC organic)	0–2.7	N/A	[63]
Corn straw	Hydrothermal carbonization and KOH activation	1229	385/0.5 (6 M KOH)	N/A	91.3% (2000)	[64]
Corn straw	KOH/ZnCl_2_ activation	2013	230/0.5 (0.5 M Na_2_SO_4_ neutral)	0–1.8	90.3% (5000)	[65]
Corn stalks	KOH activation	408	125/0.1 (6 M KOH)	0–1	88% (2000)	[66]
			62/0.1 (1 M LiPF_6_ Organic)	0–2	81% (2000)	
Corn stalk	FeCl_3_/H_2_O_2_ pretreatment and KOH activation	1842	342/0.5 (6 M KOH)	N/A	N/A	[67]
Corn cob	KOH activation	781	326/0.5 (6 M KOH)	N/A	N/A	[68]
B. Directly carbonized biomass-derived carbon systems						
Borassus flabellifer fiber	Carbonization under N_2_	46.3	45.76/1 (1 M KOH)	0–1	96.9% (500)	[69]
European hornbeam	Carbonization/ Cavitation	614	24/0.25 (1 M H_2_SO_4_)	0–0.8	100% (10,000)	[53]
Cashew nut	Self-sustained gasification (velocity 5 cm s^−1^)	14	186/0.5 (6 M KOH)	0–1	137% (10,000)	[70]
	(Velocity 20 cm s^−1^)	28	133/0.5 (6 M KOH)		123% (10,000)	
Corn straw	Facile direct carbonization under Ar	7.8	53.8/1 (1 M KOH)	0–1.5	93.7% (30,000)	This work

Note: N/A indicates that the corresponding data were not available or not reported in the referenced literature.

## Data Availability

The original contributions presented in this study are included in the article/Supplementary Material. Further inquiries can be directed to the corresponding authors.

## Supplementary Materials

The following supporting information can be downloaded at https://www.mdpi.com/article/10.3390/ma19173741/s1, Figure S1: Cumulative surface area as a function of pore width (BJH method); Figure S2: NLDFT pore size distribution of CS-600, CS-800, and CS-1000 in the micropore and small-mesopore region (0–10 nm) derived from N_2_ adsorption–desorption analysis; Figure S3: Time-dependent deionized-water contact-angle variation of CS-600, CS-800, and CS-1000 samples measured at 0, 3, 5, 8, and 10 min; Figure S4: Element distribution image of the CS-800 sample; Figure S5: (a) CV curves measured at different operating voltages at 5 mV s^−1^, (b) CV curves of the CS-800 electrode at different scan rates of 100 to 800 mV s^−1^, and (c) GCD curves collected over different current densities; Figure S6: Electrochemical performance of the CS-600 electrode in a three-electrode configuration: (a) CV curves at various scan rates and (b) GCD curves at different current densities; Figure S7: Electrochemical performance of the CS-1000 electrode in the three-electrode configuration: (a) CV curves at various scan rates and (b) GCD curves at different current densities; Figure S8: Deconvoluted voltammograms illustrating capacitive and diffusive regions scanned at (a) 40, (b) 60, (c) 80, and (d) 100 mV s^−1^; Figure S9: CV curves of (a) CS-600, (b) CS-800, and (c) CS-1000 at different scan rates, and (d) determination of the b-value from CV analysis based on the linear relationship between the plot of log(*i*) vs. log(*v*); Table S1: Raman and XPS structural parameters of CS-X materials.

## Abbreviations

The following abbreviations are used in this manuscript:

CS	Corn straw-derived carbon
SCs	Supercapacitors
SSC	Symmetric supercapacitor
EDLC	Electric double-layer capacitance
KOH	Potassium hydroxide
ZnCl_2_	Zinc chloride
H_3_PO_4_	Phosphoric acid
PVA	Polyvinyl alcohol
H_2_SO_4_	Sulfuric acid
Na_2_SO_4_	Sodium sulfate
HNO_3_	Nitric acid
N	Nitrogen
S	Sulfur
O	Oxygen
C	Carbon
P	Phosphorus
PVDF	Polyvinylidene difluoride
Super-P	Conductive carbon black
Ar	Argon
N_2_	Nitrogen gas
DI	Deionized water
HCl	Hydrochloric acid
XRD	X-ray diffraction
FT-IR	Fourier transform infrared
TGA	Thermal gravimetric analysis
RT	Room temperature
BJH	Barrett–Joyner–Halenda
BET	Brunauer–Emmett–Teller
SSA	Specific surface area
SEM	Scanning electron microscope
TEM	Transmission electron microscope
SAED	Selected area electron diffraction
EDS	Energy-dispersive X-ray spectroscopy
EPR	Electron paramagnetic resonance
XPS	X-ray photoemission spectroscopy
NLDFT	Non-localized density functional theory
CV	Cyclic voltammetry
GCD	Galvanostatic charge/discharge
EIS	Electrochemical impedance spectroscopy
CO_2_	Carbon dioxide
CO	Carbon monoxide
H_2_O	Water
SiO_2_	Silicon dioxide
SiO_x_	Silicon oxide
