# Supplementary material for: Biomass-Derived Carbon Electrodes with Defects and Porosity Prepared via Regulated Carbonization Temperature for Supercapacitors
Source: Materials (Basel). 2026 Sep 3;19(17):3741. doi: 10.3390/ma19173741 (PMC13567600; doi:10.3390/ma19173741)
Supplement: Supplementary file 1 [file materials-19-03741-s001.zip › materials-4317154-supplementary.pdf]

Biomass-Derived Carbon Electrodes with Defects and Porosity Prepared via Regulated Carbonization Temperature for Supercapacitors

Tserenlkham Byambadorj [1,2], Jiawei Zhang [1,], Xuzhen Lu [1], Yuehui Wang [1], Fan Wang [1], Qian Liu [1], Yu Li [1,*], Minghua Chen [1]*

[1] *Key Laboratory of Engineering Dielectric and Applications (Ministry of Education), School of Electrical and Electronic Engineering, Harbin University of Science and Technology, Harbin 150080, The Republic of China; tserenlkham.b@must.edu.mn (T.B.)*

[2] *School of Power Engineering, Mongolian University of Science and Technology, Ulaanbaatar 14191, Mongolia*

* *Correspondence: jwzhang@hrbust.edu.cn (J.Z.); yu.li@hrbust.edu.cn (Y.L.); Tel.: +86-182-4611-3802 (J.Z.)*

Supplementary Materials:

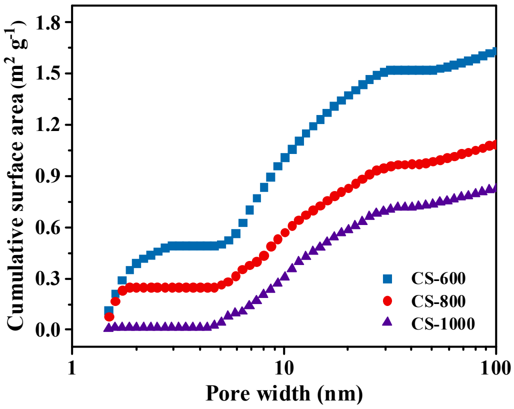

Figure S1. Cumulative surface area as a function of pore width (BJH method)

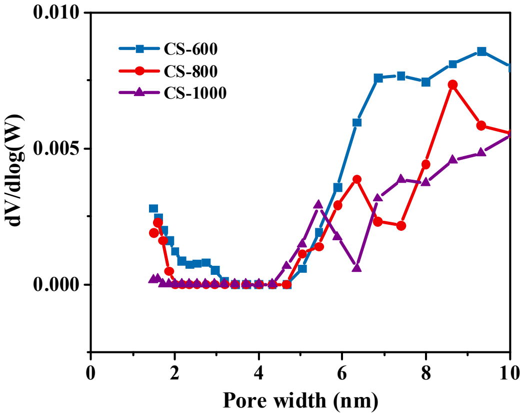

Figure S2. NLDFT pore size distribution of CS-600, CS-800, and CS-1000 in the micropore and small mesopore region (0–10 nm) derived from N_2 adsorption–desorption analysis.

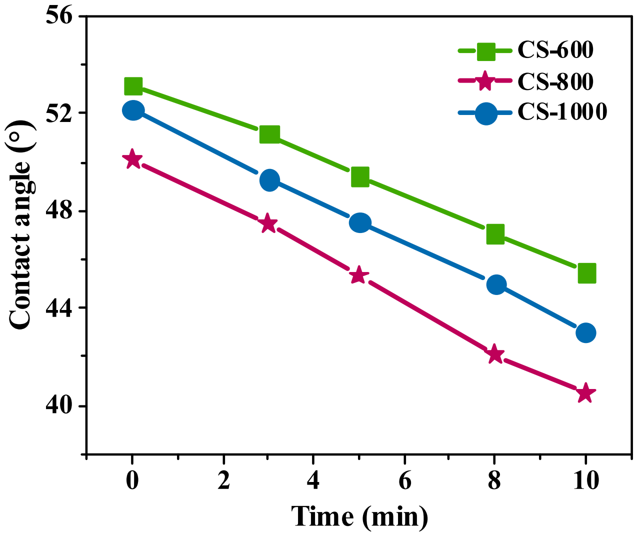

Figure S3. Time-dependent deionized-water contact-angle variation of CS-600, CS-800, and CS-1000 samples measured at 0, 3, 5, 8, and 10 min.

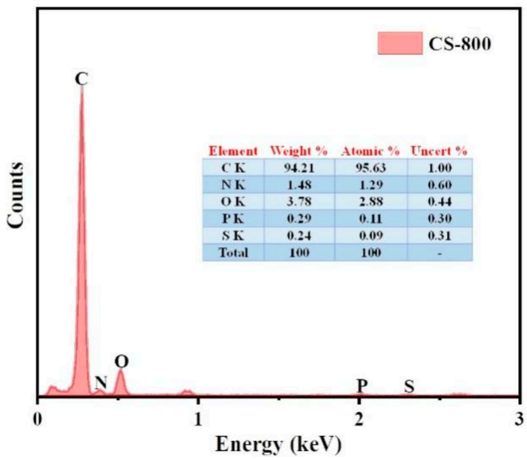

Element	Weight %	Atomic %	Uncert %
C K	94.21	95.63	1.00
N K	1.48	1.29	0.60
O K	3.78	2.88	0.44
P K	0.29	0.11	0.30
S K	0.24	0.09	0.31
Total	100	100	-

Figure S4. Elements distribution image of the CS-800 sample.

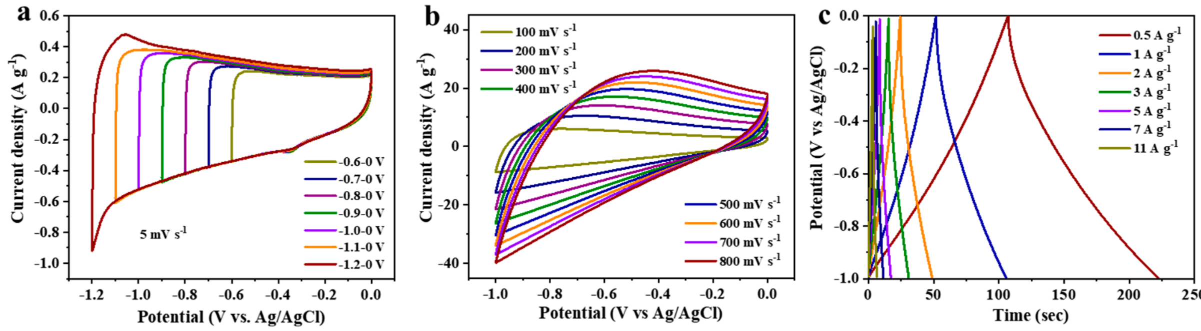

Figure S5. (**a**) CV curves measured at different operating voltages at 5 mV s^{-1}, (**b**) CV curves of the CS-800 electrode at different scan rates of 100 to 800 mV s^{-1}, and (**c**) GCD curves collected over different current densities.

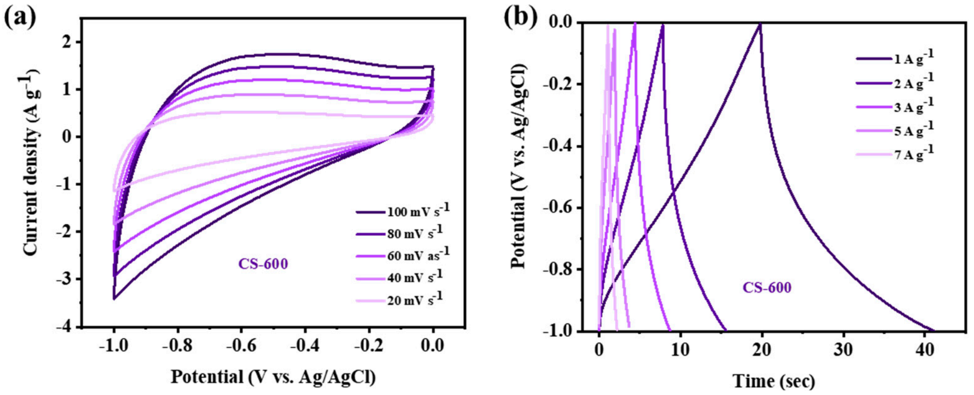

Figure S6. Electrochemical performance of the CS-600 electrode in a three-electrode configuration: (**a**) CV curves at various scan rates and (**b**) GCD curves at different current densities.

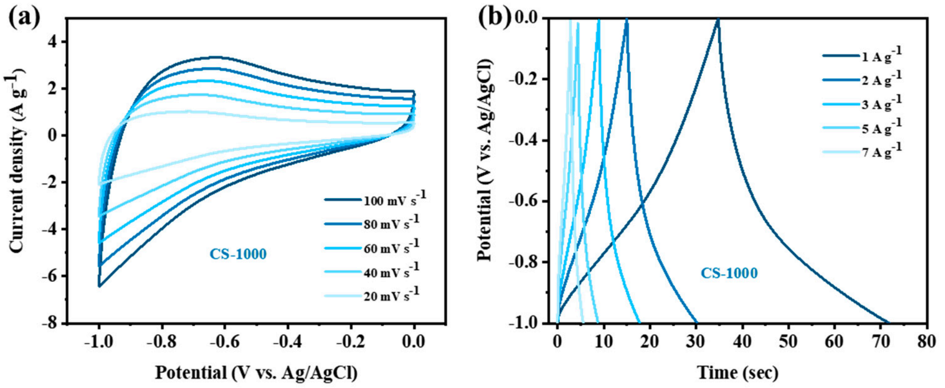

Figure S7. Electrochemical performance of the CS-1000 electrode in the three-electrode configuration: (**a**) CV curves at various scan rates and (**b**) GCD curves at different current densities.

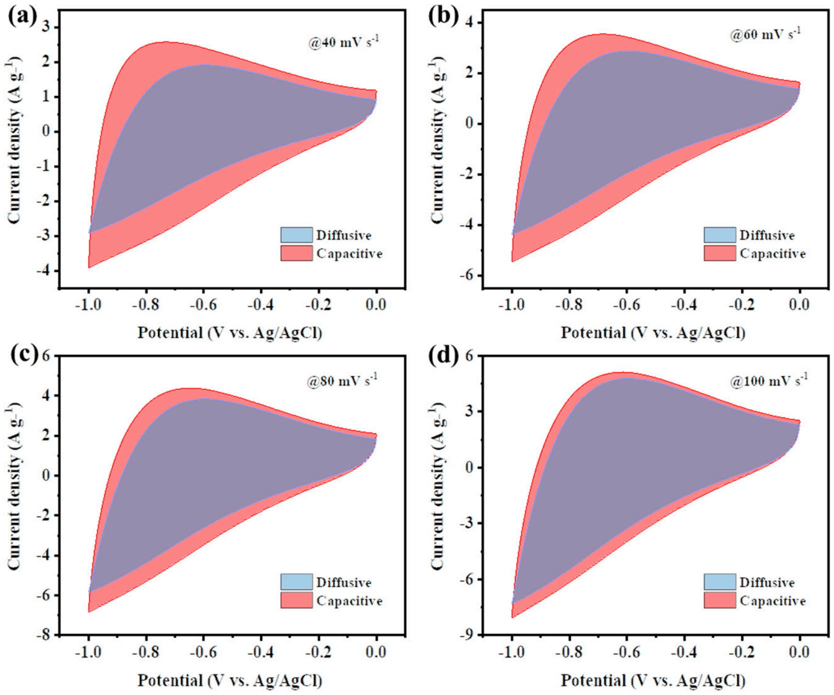

Figure S8. Deconvoluted voltammograms illustrating capacitive and diffusive regions scanned at (**a**) 40, (**b**) 60, (**c**) 80, and (**d**) 100 mV s^{-1}.

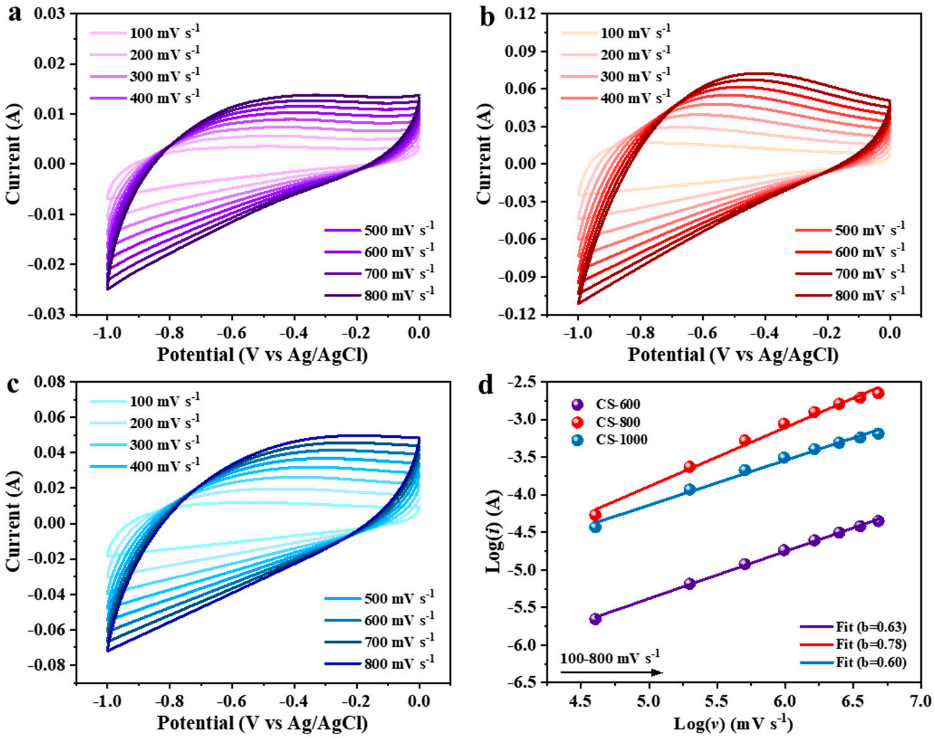

Figure S9. CV curves of (**a**) CS-600, (**b**) CS-800, and (**c**) CS-1000 at different scan rates, and (**d**) determination of the b-value from CV analysis based on the linear relationship between the plot of log(*i*) vs. log(*v*).

Table S1. Raman and XPS structural parameters of CS-X materials

Sample	I_D/I_G	C-C/C=C (%)	C-N/C-O (%)	O-C=O (%)	O (at.%)
CS-600	1.004	77.9	17.0	5.1	30.51
CS-800	1.006	78.7	16.8	4.5	28.58
CS-1000	1.011	74.0	21.4	4.6	31.08
